# Classic and recent advances in understanding amnesia

**DOI:** 10.12688/f1000research.13737.1

**Published:** 2018-03-16

**Authors:** Richard J. Allen

**Affiliations:** 1 School of Psychology, University of Leeds, Leeds, UK

**Keywords:** amnesia, memory, hippocampus

## Abstract

Neurological amnesia has been and remains the focus of intense study, motivated by the drive to understand typical and atypical memory function and the underlying brain basis that is involved. There is now a consensus that amnesia associated with hippocampal (and, in many cases, broader medial temporal lobe) damage results in deficits in episodic memory, delayed recall, and recollective experience. However, debate continues regarding the patterns of preservation and impairment across a range of abilities, including semantic memory and learning, delayed recognition, working memory, and imagination. This brief review highlights some of the influential and recent advances in these debates and what they may tell us about the amnesic condition and hippocampal function.

## Introduction

The ‘amnesic syndrome’ has a relatively high profile both in the neuropsychological literature and in popular culture. This is likely due in part to the centrality of memory in defining our place in the world and sense of self, in enabling effective everyday functioning, and of the often-striking loss of memory function in patients with amnesia, relative to healthy individuals. The term is derived from the Greek *a-* (without) - *mnesis* (memory), and at a broad level, amnesia can be defined as a profound loss of memory. The extensive impacts of this condition mean that individuals with amnesia usually require assistance in daily life.

Amnesia can be temporary or have a psychological root (for example, transient global amnesia ^1^ and psychogenic/dissociative amnesia ^2^) and is a term used in reference to memory problems in various neurological conditions (for example, amnestic mild cognitive impairment). However, the nature of these problems means that other functional and mechanistic descriptions likely apply, and so they are outside the scope of this overview. Instead, the focus is on the amnesic condition as a long-lasting or permanent disorder, emerging from an organic or neurological cause. Causes can include traumatic head injury, neurosurgery (for example, to treat severe epilepsy), anoxia/hypoxia (lack of oxygen), ischaemia, viral infection ( *Herpes simplex* encephalitis), and alcoholic Korsakoff’s syndrome. The large majority of cases represent adult-acquired memory loss, although individuals have been identified as having ‘developmental amnesia’, acquired at birth or in infancy ^3^. The neurological basis of amnesia will obviously depend on the aetiological nature and extent. Although a range of brain areas can be involved in profound memory loss (for example, the prefrontal cortex or, in the case of alcoholic Korsakoff syndrome, the thalamic/diencephalic region), neurologically derived amnesia has more commonly been associated with damage to the hippocampus specifically and to the medial temporal lobes (MTLs) more broadly. This well-established primary neurological locus means that research with patients can inform both the amnesic condition and how the hippocampus and MTL contribute to memory and cognitive function more broadly.

Understanding of amnesia has been substantially driven by case studies of patients such as HM ^4– 6^, and indeed this approach remains informative provided that robust methodological approaches are adopted ^7^. Group studies are also informative in extending beyond the individual, offering greater statistical power, and allowing identification of consistent patterns, although care must be taken when collapsing across patients with possibly heterogeneous profiles of damage and ability, and combining behavioural and imaging methodology can be useful in this regard ^8^. As noted by Clark and Maguire ^9^, although the lesion-deficit model has been dominant and studies tend to focus on how individuals with amnesia are impaired, a comprehensive picture can be obtained only by contrasting this with patterns of preservation. Debate continues regarding precisely how to characterise this profile and what this reveals about the function of neural areas putatively associated with this condition. This review aims to provide a brief overview of some of the insights and debates emerging from classic work and recent advances in the area, particularly in the context of anterograde memory difficulties displayed by individuals with hippocampal/MTL amnesia. (For more in-depth recent reviews, see, for example, 7– 10.)

## Long-term memory

Depending on aetiology and extent of lesion, individuals with amnesia will often show minimal memory following even short periods (>30 seconds) of distraction or interference, alongside relatively intact broader ability (for example, language and motor movement). Amnesia can be retrograde (that is, loss of memories acquired prior to onset) and anterograde (impairment in forming new memories), and patients typically exhibit both forms to varying extents. Severity of retrograde and anterograde loss appears to correlate ^11^, and retrograde loss follows a temporally graded pattern with greater preservation of distally acquired information, relative to memories immediately prior to onset (as described by Ribot’s law ^12^). It has been suggested that this pattern of recent retrograde loss represents disruption of long-term memory consolidation ^13^, and indeed there is some evidence that patients with amnesia show greater vulnerability to interference during consolidation ^14^.

One of the key principles generated from research with patients with amnesia is that memory is not unitary and instead can be fractionated into separable systems or abilities. Amnesic patients with MTL damage have intact procedural memory and learning (see 15 for a review), suggesting a set of implicit or non-declarative memory systems that are distinct from explicit or declarative memory (for example, 16, 17, but see 18 for an alternative interpretation). They may also have relatively preserved semantic (that is, factual) knowledge for information acquired prior to onset ^19^. However, controversy exists regarding whether new semantic information can be acquired post-onset. Patients often show considerable impairments in memory for novel facts about news events and famous individuals (for example, 20). On the other hand, work with developmental amnesia has shown that such patients indeed have been able to acquire semantic knowledge over time, although lab studies suggest that this may proceed more slowly than in healthy controls ^3, 21^. In contrast, episodic memory (that is, memory for events grounded in time and space) is consistently impaired in both (temporally graded) retrograde and anterograde form ^22^. Indeed, these episodic memory difficulties may at least partly account for why patients with amnesia struggle with acquiring new semantic knowledge ^23^.

Memory deficits in patients with amnesia appear to be particularly apparent when assessed via recall, compared to relatively reduced impairments on tests of recognition ^21, 24– 26^. For example, Patai *et al*. ^25^ assessed 29 patients with relatively selective hippocampal damage on the recall and recognition subtests of the Doors and People Test ^27^ and found larger recall deficits relative to recognition. Moreover, hippocampal volume correlated with recall performance but not with recognition. Distinctions between recall and recognition have been mapped onto experiences of recollection and familiarity; individuals with amnesia have problems with recollection (on which recall is dependent) because the MTL underlies recollection, but they remain able to make recognition judgements based on intact feelings of familiarity (for example, 28, 29). Following this, a more precise and detailed two-process approach ^30, 31^ argues that the hippocampus plays a critical role in recollection, with parahippocampal regions supporting familiarity-based recognition judgements. This implies that the recall-recognition dissociation will be more dramatic in individuals with highly selective hippocampal damage. In contrast, broader MTL damage leads to substantial deficits in both recall and recognition. Although neuropsychological and imaging data provide some support for this ^25, 31^, it is by no means universally accepted. Other work with patient groups rejects a clear recall-recognition dissociation in amnesia ^32, 33^. For example, Smith *et al*. ^34^ found widespread recognition difficulties in groups of patients with either selective hippocampal and broader MTL lesions, with the former group intact on only one sub-category of test (immediate recognition for faces). The authors argued that recognition judgements draw on item-list associations, and so (as with other forms of associative processing) even amnesic patients with selective hippocampal damage show impairment.

Somewhat reduced recognition accuracy in selective hippocampal patients, relative to controls, should also be predicted by the dual-process account of recognition, which states that both recollection and familiarity contribute to recognition judgements in typical memory functioning ^35^. These processes can be independently indexed by asking participants to make recognition-based metacognitive judgements regarding whether they remember (R) encountering an item earlier or simply know (K) that it had been previously presented, a distinction which maps onto recollection and familiarity respectively (and parallels the fractionation of episodic and semantic memory). As would be predicted on the basis of the relative absence of recollective experience, hippocampal amnesic patients have been shown to produce reduced rates of R estimates ^26^. Similarly, although the developmental amnesic individual Jon reported R judgements, he seemed unable to understand the R-K distinction ^21^, showed no brain activation differences between such judgements ^36^, and was unable to provide recollective justifications for why he made R, K, or G (guess) categorizations ^37^.

Although there is little doubt that amnesia involves severe deficits in delayed recall, debate continues as to the nature, extent, and causes of their possible difficulties in delayed recognition. Different studies have identified and emphasized patterns of relatively impaired or intact performance in various groups of patients with amnesia, and the overall picture appears to be that recognition deficits are often apparent compared with typical controls but are not as severe as those seen in recall. This may reflect, in part, the preserved ability to make familiarity-based recognition judgements, alongside deficits in recollective experience; impaired recognition performance will be observed when familiarity alone is not sufficient to adequately support performance. Nevertheless, notes of caution remain when contrasting relative patterns of recall and recognition across studies. Given the possible contributory roles played by distinct MTL structures (for example, 31), it is important that the lesion patterns of individual patients be clearly defined, as hippocampal and extra-hippocampal damage may lead to differing patterns of impairment. Furthermore, care must be taken to consider issues such as task difficulty and data sensitivity when comparing recall and recognition measures (for example, 25), as recall tends to be a more difficult task and produces reduced accuracy levels in healthy individuals as well as patient groups ^38^.

## Working memory

Classic work on amnesia indicates that, although delayed (long-term) episodic memory is impaired, patients show intact immediate or working memory (that is, the ability to “hold a limited amount of information temporarily in a heightened state of availability for use in ongoing information processing” ^39^). HM, for example, demonstrated preserved digit recall provided that he was not distracted and sequence length did not exceed his immediate memory capacity ^6, 40^. Similar intact performance levels on a range of verbal and visuospatial working memory tasks have been repeatedly observed in other patients (for example, 22, 41). Patients with amnesia also demonstrate apparently intact ability to use prior knowledge to facilitate working memory performance (for example, by showing superior memory for sentences over random word lists ^42, 43^ and for digits when embedded in familiar ‘keypad’ configurations ^43, 44^).

However, it has been argued that broader abilities attributed to the hippocampus may lead to amnesic patients showing deficits even in working memory tasks that require these abilities. For example, the hippocampus plays a key role in spatial processing ^45^, and indeed patients with amnesia show deficits in some (but not all ^46, 47^) aspects of spatial navigation ^48^ and allocentric memory ^49^. Memory for associations or relational bindings between features of a stimulus or event is also thought to have a critical hippocampal component ^50– 52^. In regard to working memory, tasks that emphasize relational and allocentric spatial processing (regarding the relative relationship between objects in the environment, independent of the viewer) and minimize egocentric contributions indicate impaired performance in hippocampal/MTL patients, even at retention intervals of only a few seconds ^53, 54^. Similarly, short-term memory for relational binding between object and location, scene, or object has also been shown to be impaired and error-prone in MTL amnesic patients ^55– 59^.

Evidence is mixed, however, with other patients (for example, Jon) showing intact immediate memory for binding between shape and color ^42^, and between object and location, alongside impaired delayed memory for the same material ^60^. Along similar lines, Jeneson *et al*. ^61– 63^ have observed intact working memory performance at short delays and small memory loads in hippocampal/MTL amnesics (see also 64), and deficits consistently emerge only when these task factors are increased and the limits of working memory are exceeded. Consistent with this, a recent comparison of relational memory for word pairs indicated that patients with amnesia were able to recall these relations in an immediate test but (unlike controls) not in a delayed test 25 minutes later ^65^. It has been argued, on the basis of such results, that apparent evidence for immediate/working memory deficits on such tasks in individuals with hippocampal/MTL amnesia reflects conditions in which working memory capacity is exceeded and support from long-term memory is required ^61^.

Despite this, it remains possible that specific forms of temporary memory processing are indeed impaired in amnesic patients with hippocampal damage. It may be that relational binding (that is, memory for the relationship between object and context) is impaired but conjunctive memory (that is, for the binding between features of unitary objects, such as shape and color) is intact ^59, 66^. Additionally, Yonelinas ^67^ has argued that the hippocampus supports high-resolution binding across perception, working memory, and long-term memory. Indeed, most research on working memory in amnesia has used categorical tasks not explicitly designed to index degradation of representational fidelity, although the limited number of studies using precision-based methods with patients with amnesia have produced mixed results ^10^. Further research is clearly required here.

While these debates continue, they also illustrate how distinctions between types of task are not necessarily absolute in terms of the underlying mechanisms they may index and that, as noted by Eichenbaum ^5^, hippocampal activation involved in longer-term memory retention is likely to begin during initial encoding of experiences. As such, at a functional level, individuals with amnesia may experience difficulties in memory tasks even when tested with relatively short delays.

## Visualization, imagination, and scene construction

Amnesia is often referred to as a ‘global’ disorder (that is, with memory deficits that apply across verbal and visuospatial information). It is nevertheless important when assessing memory capability of such patients that a variety of tasks be administered, tapping memory for different types of information. Furthermore, there is some limited evidence that hippocampal damage may have a greater impact on visualization and visuospatial memory. For example, Patai *et al*. ^25^ found some evidence that hippocampal amnesic patients demonstrated greater memory problems in the visual domain, relative to verbal memory. Similarly, within verbal memory paradigms, there is some evidence that patients with amnesia show greater impairment in concrete relative to abstract words ^68^, suggesting that they may be less able to use imagery to supplement verbal memory ^69^.

These findings are in line with the greater emphasis that theories of hippocampal function often place on visuospatial processing ^9^. For example, they speak to the theoretical framework developed by Maguire *et al*. ^69– 71^ proposing that hippocampal amnesic patients have imagination and scene construction deficits that emerge across a range of tasks requiring the generation of coherent internal scenario or scene. Following initial reports of well-known patients such as HM and KC experiencing problems in imagination and projection into the future ^72, 73^, such deficits have subsequently been demonstrated experimentally in different groups of patients ^74– 76^. These deficits typically contrast with intact ability to imagine isolated objects and fragmented scenes, and to describe pictures and generate related narratives, in line with the possible centrality of scene construction. Similarly, problems in future thinking particularly relate to the imagination of detailed and spatially coherent future events rather than to temporal projection more generally ^77– 79^. Patients with amnesia also appear to show difficulties in deciding whether scenes are spatially coherent or impossible ^80^, and in carrying out spatially based counterfactual thinking ^81^, compared with controls. Finally, evidence of perceptual deficits in tasks requiring discrimination between spatial scenes has been observed in selective hippocampal damaged patients, whereas deficits in both spatial and non-spatial discrimination are apparent in patients with broader perirhinal/MTL damage ^82^. It should be noted that, although the preponderance of evidence indicates imagination and scene construction deficits, some patients do demonstrate performance similar to that of control participants in such tasks ^83, 84^. This may reflect, in the case of the patients of Squire *et al*. ^84^, the availability of relatively intact autobiographical memory in that group ^9^ or the use of intact semantic memory to support scene construction in the case of Mullally *et al*. ^83^.

## Concluding remarks

Although there is a consensus, at a broad level, about what the amnesic condition represents, debate continues regarding the precise patterns of preservation and impairment and what may be identified as being primary deficits or secondary/resulting features of the condition. Early studies of amnesia played a key role in the development of structural approaches to memory, drawing distinctions between systems based on dimensions such as content, capacity and temporal duration. Recent theorizing has increasingly adopted function- and process-oriented perspectives, in part through the influence of neuroimaging research with healthy and clinical populations. Within this context, it remains to be seen whether theoretical views that emphasize, for example, consolidation, recollection, relational memory, fidelity of processing, or scene construction provide the most comprehensive primary account of the patterns of impairment (and preservation) that are typically observed or whether it is a condition that is better characterised as a grouping of multiple, separable (and comorbid) deficits. Explanations at the micro level may also be usefully integrated at the macro level in order to develop systems-based structural frameworks within which patterns of preservation and deficit, and associated brain dynamics, across a broad range of tasks might be conceptualised (for example, 85). 

Difficulties with theorizing regarding amnesia were noted over 20 years ago by Mayes and Downes ^86^. Indeed, although great progress has been made in the development of new perspectives on the condition and the integration of behavioural and imaging techniques in this context, many of these basic issues remain. The multidimensional heterogeneity that is apparent between individuals and groups in terms of aetiology, locus and extent of lesion (and corresponding residual intact tissue), and degree of post-onset compensation/neural organization remains a source of challenges for drawing interpretations that can be generalized beyond single studies. Methodological differences between studies are also important to consider as a caveat to interpretation, as are issues of task validity and the extent to which patients may be able to supplement otherwise impaired performance through the use of strategy or intact cognitive abilities. Finally, the debates in the literature have often been framed in terms of the theoretical implication for hippocampal function, but they should also consider what the outcomes of these debates might mean for diagnostic technique and for understanding (and ameliorating) the impacts of the amnesic condition on a patient’s quality of life and everyday functioning. 

## Abbreviations

MTL, medial temporal lobe.
